# High-quality, ecologically sound remediation of acidic soil using bicarbonate-rich swine wastewater

**DOI:** 10.1038/s41598-017-12373-9

**Published:** 2017-09-19

**Authors:** Cheng Qilu, Wu Xueling, Xu ligen, Lin Hui, Zhao Yuhua, Zhou Qifa

**Affiliations:** 1 College of Life Sciences, Zhejiang University, Hangzhou, 310058 China; 2 College of Agriculture and Biotechnology, Zhejiang University, Hangzhou, 310058 China; 3 Institute of Environment, Resource, Soil and Fertilizer, Zhejiang Academy of Agricultural Sciences, Hangzhou, China

## Abstract

The swine industry in China is experiencing a wastewater crisis. In this work, we found that swine wastewaters were particularly high in bicarbonate (1.52–9.25 g/L, mean = 5.68 g/L, *n* = 42). The high level of bicarbonate may add to the pollution load during discharge. We therefore suggest a new method for bicarbonate-rich wastewater remediation in acidic soil. In our laboratory irrigation experiments, wastewater irrigation efficiently increased the pH and decreased the exchangeable aluminum in the acidic soil. Furthermore, the wastewater method efficiently remediated the entire soil body, while lime application remediated only a portion of the topsoil. Wastewater irrigation also improved soil fertility (e.g., by increasing the phosphorus availability in acid soil).

## Introduction

China is the largest swine producer in the world^1^. However, the Chinese swine industry is experiencing a wastewater disposal crisis owing to the launching of the “Environmental Protection Storm” by the Chinese government. In Zhejiang Province alone, about 45 thousand farms have been forced to close since 2013^2^.

The large volume^3^ and high concentrations of chemical oxygen demand^4^ and ammonia^4,5^ in swine wastewater pose an economic and technical challenge regarding treatment. For example, 6.0 billion tons of swine wastewater are discharged annually in China without appropriate disposal^4^. Wastewater reuse is an emerging strategy that can confer a variety of environmental benefits^6^. Generally, water and nutrients are regarded as the only useful wastewater components. Pollutants and nutrients have been the focus of previous investigations on swine wastewater composition^3–5,7,8^. In recent years, bicarbonate in wastewater has been recognized to facilitate algal blooms^9^ and cause toxicity in some aquatic organisms^10,11^. However, if swine wastewater with high ammonia concentrations also possesses sufficiently high levels of bicarbonate, it can be suitable for acidic soil remediation for the following two reasons. (1)The bicarbonate in wastewater neutralizes the protons in the soil (Equation 1), and (2) reacts with Al^3+^ in the soil through a bi-hydrolization reaction (Equation 2) after the proton and Al^3+^ in the acidic soil are released by wastewater extraction and wastewater cation (e.g., NH_4_^+^, Ca^2+^) exchange:

1 $${\rm{Acidic}}\,{\rm{soil}}+{\rm{Wastewater}}\to {{\rm{H}}}^{+}+{{{\rm{HCO}}}_{3}}^{-}={{\rm{H}}}_{2}{\rm{O}}+{{\rm{CO}}}_{2}$$

2 $${\rm{Acidic}}\,{\rm{soil}}+{\rm{Wastewater}}\to {{\rm{Al}}}^{3+}+3{{{\rm{HCO}}}_{3}}^{-}={\rm{Al}}{({\rm{OH}})}_{3}+3{{\rm{CO}}}_{2}$$

Herein, we investigated the characteristics of swine wastewater from 30 swine farms in Zhejiang Province and explored the feasibility of applying swine wastewater for acidic soil remediation.

## Results

### Wastewater bicarbonate levels

Bicarbonate concentrations were particularly high (1.52–9.25 g/L, mean = 5.68 g/L, *n* = 42; see Table S1), and the wastewater pH was higher than 7.4 (7.47–9.12, mean = 8.42, *n* = 42; see Table S1).

### Effects of bicarbonate-rich wastewater application on pH and exchangeable Al in acidic soils

**Figure 1:**
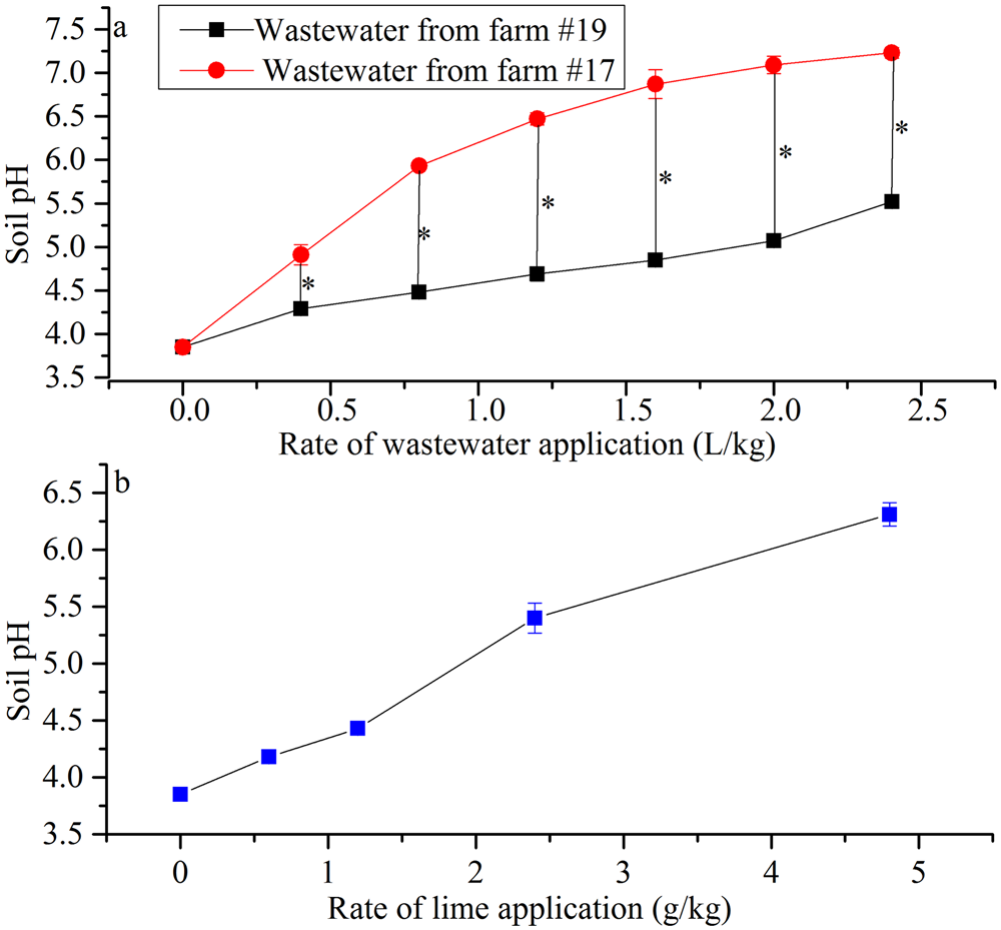
The effects of (**a**) wastewater and (**b**) lime application on the acidic soil pH. Error bars over the symbols represent the standard error of the means (n = 3), while * represents statistically significant difference at P ≤ 0.05.

**Figure 2:**
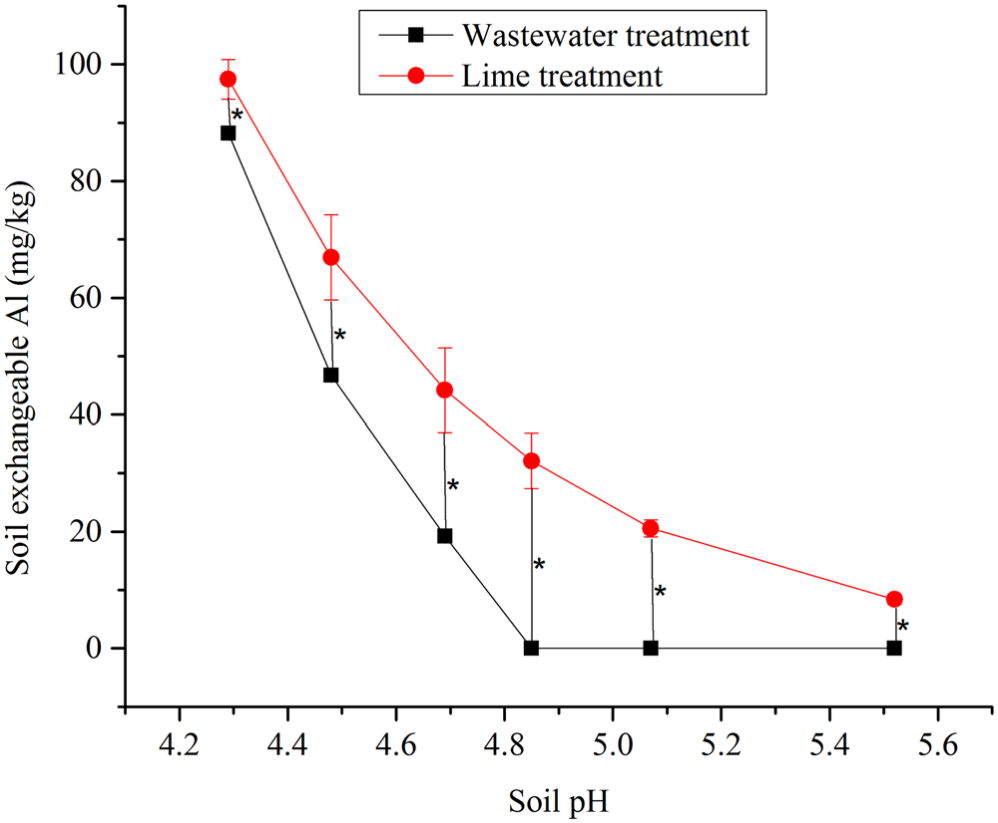
The soil exchangeable Al content versus the soil pH in lime- and wastewater- treated acidic soil. The soil exchangeable Al and pH data were measured for the wastewater treatment; for the lime treatment, the exchangeable Al corresponding to a pH consistent with the wastewater treatment was calculated according to best-fit equations between the measured pH and the measured exchangeable Al. The best-fit equation was: Soil exchangeable Al = 509735 × EXP(−1.996 × pH), *R* ^2^ = 0.9827, *n* = 12. Error bars over the symbols represent the standard error of the means (*n* = 3), while * represents statistically significant difference at *P* ≤ 0.05.

In laboratory irrigation experiments, the soil pH increased and soil exchangeable Al decreased with increasing application rates of both lime and wastewater. The pH increased from 3.85 to approximately 5.5 after applying 2.40 g lime or 2.40 L of wastewater sample from farm #19 per kilogram of soil (Fig. 1a,b). Bicarbonate concentrations in the wastewater sample from farm #17 were 381% of those contained in wastewater from farm #19. Consequently, the soil irrigated with wastewater from farm #17 had a significantly higher (P < 0.05) pH than that irrigated with wastewater from farm #19 at all irrigation rates. Moreover, the pH in soil irrigated with 0.4 L/kg of wastewater from farm #17 was close to that measured in soil irrigated with 1.6 L/kg of wastewater from farm #19 (Fig. 1a) because of the similar doses of bicarbonate. These results indicate that bicarbonate was the dominant neutralizing agent in the acidic soil–wastewater mixture. The soil exchangeable Al decreased from 201.6 mg/kg to zero with the application of 4.80 g/kg lime or 1.60 L/kg of wastewater from farm #19. Furthermore, at a given pH, the exchangeable Al in wastewater-irrigated soil was significantly (P < 0.05) lower than that in the limed soil, and the pH of wastewater-irrigated soil with zero Al was 1.46 units lower than that in the limed soil (Fig. 2).

### Degree of lime contact in the soil

**Figure 3:**
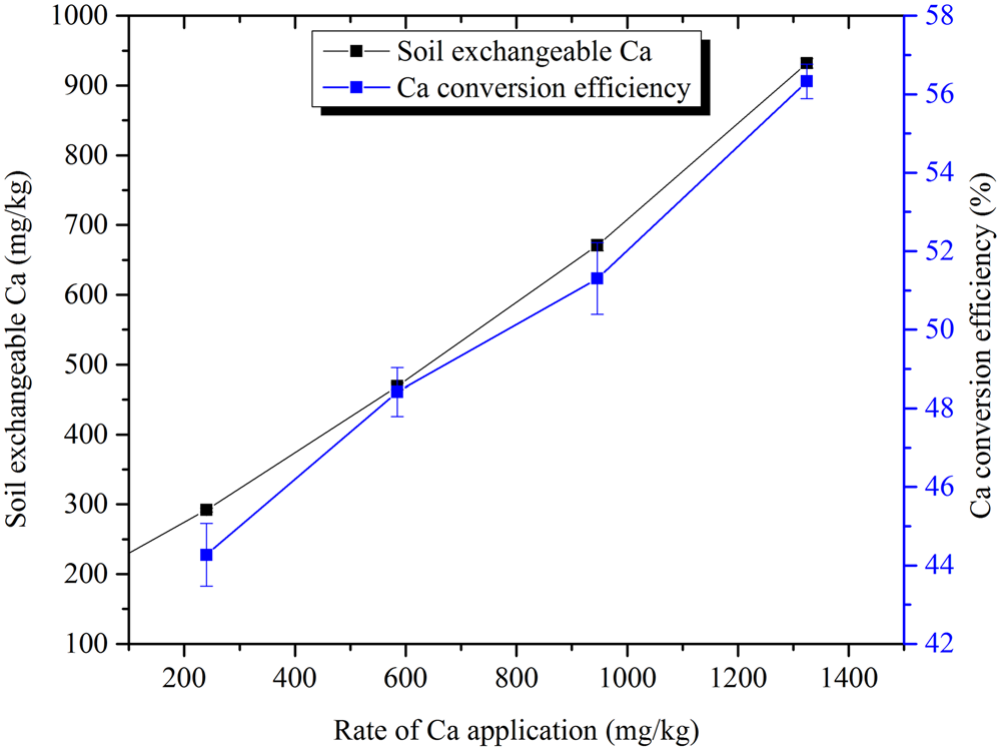
The soil exchangeable Ca content and Ca conversion efficiency in acidic soil with different rates of lime application. Error bars over the symbols represent the standard error of the means (*n* = 3).

The Ca conversion efficiency was only 56.3% at the highest liming rate. Further, both the soil exchangeable Ca and the Ca conversion efficiency increased with the liming rate (Fig. 3), indicating that the degree of lime contact in the soil was low and increased with the liming rate. These results reveal that the lime–soil mixing was not sufficient even under the vigorous stirring and saturated water conditions experienced during the experiments.

### Effects of wastewater and lime on pH in different soil layers

Lime application was effective in soil mitigation only in SL1 (0–3 cm) and SL2 (3–6 cm), but wastewater application was effective in all layers (0–30 cm; Fig. S1). The differences in pH between the top and bottom layers were 3.24 units and 0.58 units for the lime treatment and wastewater treatment, respectively (Fig. S1).

### NH_4_-N and P adsorption in acidic soil

Both the NH_4_-N adsorption isotherm and the P adsorption isotherm (Fig. S2) for the wastewater were well simulated with the Langmuir equation, achieving *R* ^2^ = 0.9968 and *R* ^2^ = 0.9999, respectively. The maximum adsorption capacity (Q_m_) values were 327.4 mg/kg and 782.6 mg/kg for NH_4_-N and P, respectively. These results indicate that the acidic soil efficiently retained the N and P present in the wastewater.

## Discussion

Swine wastewater consists primarily of residual drinking water, rinse water, urine, solid manure, and residual feed. A fattening pig can generate 8.0 kg of wastewater daily, as well as 2.0 kg of solid manure and 3.3 kg of urine^12^. Therefore, swine wastewater is often high in both biosolids and dissolved organic matter, which are rich in carbon (Table S2). Organic carbon can be digested into CO_2_ by a number of bacterial species in solid manure and wastewater, and this CO_2_ can then be converted into HCO_3_^−^ under alkaline conditions. Furthermore, the urine, drinking water, and rinse water had considerable bicarbonate concentrations (Table S2). Additionally, swine feed may contain CaCO_3_. Therefore, it is reasonable that swine wastewater contains high levels of bicarbonate. Bicarbonate is also widely present in natural water; the mean bicarbonate concentration in the three main freshwater rivers in China (the Yangtze, Yellow, and Pearl Rivers) was reported to be 142 mg/L^13^, while the mean concentration in 77 rivers in North America, South America, Asia, Africa, Europe, and Oceania was 146 mg/L^11^. Therefore, the bicarbonate concentration in the swine wastewater is ten times higher than that in typical freshwater systems. This high level of bicarbonate could add to the pollution load of the discharged wastewater.

Acidic soils are widely distributed in China and other counties^14^, occupying approximately half of the total arable land area^15,16^. Cropland soil acidification has intensified in recent decades^15,17^. Al toxicity is the dominant factor limiting crop growth in acid soil^16^. Currently, liming is the most common and practical strategy to combat soil acidification and acidity. In contrast to the easily released H^+^ in acidic soil, Al^3+^ in the exchange complex^18^ can only be released by Ca^2+^ replacement when lime is in contact with soil. Therefore, the remediation efficiency for Al toxicity is dependent on the degree of lime contact in the soil, which is determined by the liming rate and degree of lime mixing in the soil. As a) soil has substantial spatial heterogeneity because of its complicated composition and large variability in particle size, and b) lime has a very low mobility in soil, the degree of lime mixing in soil can be low. In particular, the degree of lime mixing in soil can be far lower in field situations than under experimental conditions. The results of this study highlighted the low degree of lime contact in soil, and indicated that liming could limit the quality of remediation. In the field, lime may only remediate a portion of the topsoil, while wastewater can efficiently remediate the entire soil body. Swine wastewater irrigation is simple and convenient, can contribute to solving the wastewater discharge problem in swine production, and help remediate acid soil, resulting in soil of high quality. Moreover, Swine wastewater irrigation can be practiced at a cost as low as that of wastewater distribution. A 10,000-head swine farm can generate approximately 20,000 tons of wastewater annually, which can be applied at a rate of 0.8 L/kg to approximately 6 ha of acidic soil for remediation of the top 30-cm soil layer. Furthermore, the high concentrations of organic carbon and nutrients (e.g., N and P) in wastewater could be beneficial for soil fertility. In particular, P addition and pH adjustment because of wastewater irrigation could substantially increase the P availability, as acidic soil is generally deficient in P. Generally, there are very low concentrations of persistent contaminants (e.g., heavy metals, see Table S2) in swine wastewater^19^. However, further work is needed to assess the risks associated with wastewater irrigation (e.g., pathogen load and potential groundwater pollution). Soil remediation with suitable wastewater is a novel strategy than can drive a range of benefits.We suggest that the environmental protection authorities should adopt a policy encouraging swine wastewater utilization instead of closing swine farms to eliminate swine wastewater generation.

## Methods

### Wastewater sampling

A total of 1 L of swine wastewater (fresh swine effluent from the outlets of pigsties and/or anaerobically digested effluent) was collected at each farm in an acid-rinsed polyethylene bottle between November 2016 and July 2017. For the wastewater samples from farms #17 and #19, the sample volume was 25 L each. Fresh swine urine, drinking water, and rinse water were also sampled at farm #17 with an acid-rinsed polyethylene bottle. All samples were transported within 12 h in an icebox to the laboratory, separated into 250 mL subsample aliquots in acid-rinsed polyethylene bottles, and stored at −4 °C.

### Measurement of wastewater properties

Wastewater pH was measured with a PHB-4 model pH meter (INESA CO., Shanghai). Wastewater samples were also analyzed for bicarbonate, dissolved organic carbon (DOC), NH_4_-N, NO_3_-N, PO_4_-P, and heavy metal concentrations according to the Chinese State Environmental Protection Agency Standard Methods^13^. The wastewater samples were first centrifuged at 7,000 rpm for 2 min, after which the collected supernatant was filtered through a 0.45 μm cellulose membrane. The filtrates were analyzed for HCO_3_^−^, Cl^−^, and SO_4_^2−^ (using the methods described by Kozaki *et al*.^20^ via ion chromatography using a Dionex ICS-1500 ion chromatography system with a IonPac AS11-HC 4 × 50 mm column, SpectraLab Scientific Inc., Canada), DOC (using a multi N/C 3100 Analyzer, Analytik Jena AG, Germany), NH_4_-N (using the Nash-reagent spectrophotometric method), NO_3_-N (using the phenoldisulfonic acid method), and PO_4_-P (using the molybdenum–antimony anti-spectrophotometric method). In addition, K, Ca, Mg, Fe, Cu, Zn, Al, Mn, Cd, Pb, Ni, As, Cr, Co, Sr, and Sn concentrations were determined via inductively coupled plasma mass spectrometry (ICP-MS; Agilent7500a, Agilent Technologies, USA). The solid suspension (SS) was determined by filtering 100 mL of wastewater through a pre-weighed Whatman filtration paper, and then weighing the filtration paper and the solid particles after drying to a constant weight at 7 °C. The C and N contents of the SS were determined by a CHNOS elemental analyzer (Elementar, Germany) after the dried SS was ground and passed through a 0.149 mm sieve.

### Laboratory irrigation experiments

#### Irrigation experiment 1

Irrigation experiment 1 was conducted at our lab between February and April 2017. The room was kept a constant temperature of 25 °C. The acidic soil consisted of inceptisol obtained from a tea farm in Hangzhou, China. The soil was air-dried, ground, and passed through a 2 mm sieve. A 1.2 kg soil sample was placed in a polyethylene pot (height: 12.5 cm, diameter: 10.0 cm). Three treatments were applied. (1) Liming: applying lime (CaCO_3_, AR grade) at rates of 0, 0.6, 1.2, 2.4, and 4.8 g/kg, and irrigating with deionized water at rates of 0, 0.4, 0.8, 1.2, and 1.6 L/kg, respectively; (2) wastewater irrigation using the sample from farm #17: irrigating using the wastewater at rates of 0, 0.4, 0.8, 1.2, and 1.6 L/kg; and (3) wastewater irrigation using the sample from farm #19: irrigating using wastewater at rates of 0, 0.4, 0.8, 1.2, and 1.6 L/kg. Irrigations consisted of intervals in which 0.4 L/kg of irrigant was added at a time after air-drying of the soil. The soil was stirred vigorously during irrigation. The experimental design was randomized, and three replicates were performed for each sample.

#### Irrigation experiment 2

Irrigation experiment 2 was conducted at our lab between March and April 2017. This irrigation experiment employed polyethylene columns with diameters of 9.0 cm and heights of 30.0 cm. The same soil used in irrigation experiment 1 was used in experiment 2. Two treatments were applied. (1) Liming: applying lime (CaCO_3_, AR grade) at a rate of 4.8 g/kg, and irrigating with deionized water at a rate of 2.4 L/kg; and (2) wastewater application using the sample from farm #19: irrigating with the wastewater at a rate of 2.4 L/kg. Irrigations consisted of intervals in which 0.4 L/kg of irrigant was added at a time after air-drying of the soil. The experimental design was randomized, and three replicates were performed for each sample. After air-drying of the irrigated soil, the soil pH was measured in each 3 cm layer from the top downward.

### Measurement of soil properties

#### Soil pH

Soil pH was determined from a 1:1 soil/water extract shaken for 24 h, centrifuged at 14,500 rpm for 30 min, and then passed through a 0.1 μm filter to remove colloidal Al fractions. The pH was determined with a PHB-4 model pH meter (INESA CO., Shanghai, China).

#### Soil exchangeable Al and Ca

Soil exchangeable Al and Ca were determined using a KCl extraction method. The KCl concentration was 1 mol/L, and the soil-to-liquid ratio was 1:10. After mixing the soil and the KCl solution, the mixture was agitated on a reciprocating shaker at 120 rpm for 30 minutes. After a 30-minute centrifugation period, the supernatant was filtered through a 0.22 μm pore-size Millipore filter for Al and Ca measurement. Al and Ca concentrations were determined via inductively coupled plasma mass spectrometry (ICP-MS, Agilent7500a, Agilent Technologies, USA).

### Soil adsorption experiment

The soil adsorption experiment was conducted at our lab in July 2017. A 1.50 g sample of the same acidic soil used in the irrigation experiments was placed in a 50 mL centrifuge tube with either 10.0 mL of the original wastewater sampled at farm #17 in July 2017 or wastewater diluted at ratios of 1:2, 1:4, 1:6, 1:8, and 1:10. The tubes were shaken at 25 ± l °C for 24 h and then centrifuged at 7,000 rpm for 2 min, after which the collected supernatant was filtered through a 0.45 μm cellulose membrane. The filtrates were analyzed for NH_4_-N and PO_4_-P. The soil adsorption capacity for NH_4_-N and PO_4_-P was calculated based on the concentrations before and after the adsorption equilibrium. The adsorption isotherms were simulated with the Langmuir equation in Origin 9.0 (OriginLab Corporation).

### Definitions of Ca conversion efficiency and zero-Al pH

The calcium conversion efficiency (CaCE, %) is defined as the percent of applied Ca converted to soil exchangeable Ca:

3 $${\rm{CaCE}}( \% )=({\rm{Caa}}-({\rm{CaE1}}-{\rm{CaE0}}))\times 100$$

where Caa, CaE1, and CaE0 represent the Ca applied to the soil (mg/kg), the soil exchangeable Ca (mg/kg) after Ca application, and the soil exchangeable Ca (mg/kg) before Ca application, respectively.

Zero-Al pH is defined as the soil pH when exchangeable Al decreases to zero with the application of a remediation agent.

### Statistics

The means and standard deviations of the data were calculated using Microsoft Excel. A t-test was used to compare the paired means. Differences were considered significant at *P* ≤ 0.05. Statistical analyses were performed in SPSS, version 16.0 (SPSS, Inc., Chicago, USA).

## Electronic supplementary material

Supplementary Information
