# Supplementary material for: High-quality, ecologically sound remediation of acidic soil using bicarbonate-rich swine wastewater
Source: Sci Rep. 2017 Sep 19;7:11911. doi: 10.1038/s41598-017-12373-9 (PMC5605680; doi:10.1038/s41598-017-12373-9)
Supplement: Supplementary file 1 — Supplementary Information [file 41598_2017_12373_MOESM1_ESM.pdf]

High-quality, ecologically sound remediation of acidic soil using bicarbonate-rich swine wastewater

Cheng Qilu[1], Wu Xueling[1], Xu ligen[2], Lin Hui[3], Zhao Yuhua[1], Zhou Qifa[1, *]

[1]College of Life Sciences, Zhejiang University, Hangzhou 310058, China

[2]College of Agriculture and Biotechnology, Zhejiang University, Hangzhou 310058, China

[3]Institute of Environment，Resource, Soil and Fertilizer, Zhejiang Academy of Agricultural Sciences, Hangzhou, China

*Corresponding author. Email: zzzqqq@zju.edu.cn

Table S1 | The pH and the bicarbonate content in wastewater from 30 farms in Zhejiang Province, China. FSE: fresh swine effluent from the outlets of pigsties. ADE: anaerobically digested effluent.

Farm #	Scale (thousand heads)	Sample date	Wastewater type	pH	Bicarbonate (g/L)
1	2.2	Nov,2016	FSE	8.49	2.34
1	2.2	Nov,2016	ADE	8.16	2.89
2	5.5	Nov,2016	FSE	8.29	4.11
2	5.5	Nov,2016	ADE	7.76	5.21
3	4.0	Nov,2016	ADE	8.30	9.25
4	13.5	Nov,2016	FSE	8.10	4.15
4	13.5	Nov,2016	ADE	7.74	4.9
5	3.5	Nov,2016	FSE	7.88	7.75
5	3.5	Nov,2016	ADE	7.55	9.25
6	6.5	Nov,2016	ADE	7.85	2.34
7	7.3	Nov,2016	ADE	7.92	2.68
8	12.1	Dec,2016	FSE	8.79	4.93
8	12.1	Dec,2016	ADE	7.90	5.77
9	8.5	Dec,2016	ADE	7.99	3.76
10	2.6	Dec,2016	ADE	7.70	3.02
11	15.5	Dec,2016	ADE	7.60	5.51
12	3.9	Dec,2016	FSE	7.75	2.66
12	3.9	Dec,2016	ADE	7.55	3.04
13	6.0	Dec,2016	FSE	8.50	2.52
13	6.0	Dec,2016	ADE	8.30	3.03
14	22.0	Dec,2016	FSE	8.18	2.33
14	22.0	Dec,2016	ADE	8.20	3.5
15	106.0	Dec,2016	FSE	8.20	5.04
15	106.0	Dec,2016	ADE	8.30	5.73
16	2.9	Dec,2016	FSE	8.05	3.66
16	2.9	Dec,2016	ADE	8.10	4.22
17	2.5	Feb,2017	ADE	7.65	5.80
18	110.0	Feb,2017	FSE	7.69	5.08
18	110.0	Feb,2017	ADE	7.59	5.57
19	5.0	Feb,2017	ADE	7.91	1.52
20	3.2	April,2017	ADE	8.45	6.30
21	2.1	April,2017	ADE	8.20	5.45
22	181.0	April,2017	ADE	8.10	5.28
23	57.0	April,2017	ADE	8.59	5.01
24	35.0	April,2017	ADE	7.71	4.88
25	2.6	April,2017	ADE	8.50	2.18
26	2.3	April,2017	ADE	9.12	5.98
27	3.3	April,2017	ADE	8.96	8.77
28	5.1	April,2017	ADE	9.00	6.60
29	2.4	April,2017	FSE	7.47	5.03
29	2.4	April,2017	ADE	7.83	5.51
30	15.0	April,2017	ADE	9.07	7.11

Table S2 | The properties of wastewater from farm #17, drinking water, rinse water, and fresh urine. SS: solid suspension. DOC: dissolved organic carbon. The SS had a C content of 46.39% and an N content of 5.59%.

	Wastewater from farm #17	Drinking and rinse water	Fresh urine
pH	7.65	7.33	6.68
HCO_3^- g/L	5.80	0.096	1.90
SS g/L	7.66		
DOC g/L	5.47		
NH_4-N mg/L	1125.89		
NO_3-N mg/L	2.16		
PO_4-P mg/L	20.54		
K mg/L	11.02		
Ca mg/L	213.50		
Mg mg/L	2.49		
Mn mg/L	0.14		
Fe mg/L	1.26		
Cu mg/L	0.055		
Zn mg/L	0.29		
Cr mg/L	0.003		
Ni mg/L	0.07		
Pb mg/L	0		
Cd mg/L	0		
Al mg/L	0		
As mg/L	0.007		
Co mg/L	0.017		
Sr mg/L	0.07		
Sn mg/L	0.06		
Cl^- mg/L	149.60		
SO_4^{2-} mg/L	78.60		

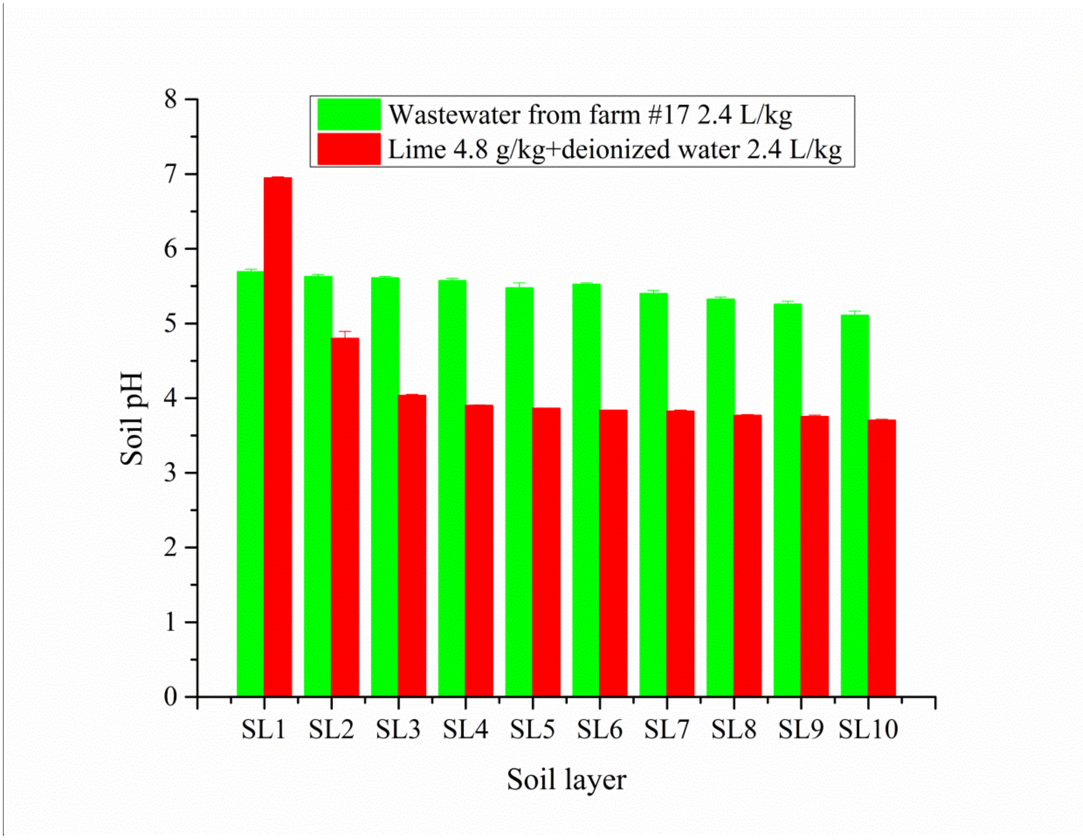

Figure S1 | The effects of lime and wastewater application on the pH in different soil layers. Data are means ± S.D. (n=3). SL1, SL2, SL3, SL4, SL5, SL6, SL7, SL8, SL9, and SL10 represent the soil layers at 0-3, 3-6, 6-9, 9-12, 12-15, 15-18, 18-21, 21-24, 24-27, and 27-30 cm in a column of 30 cm height, respectively.

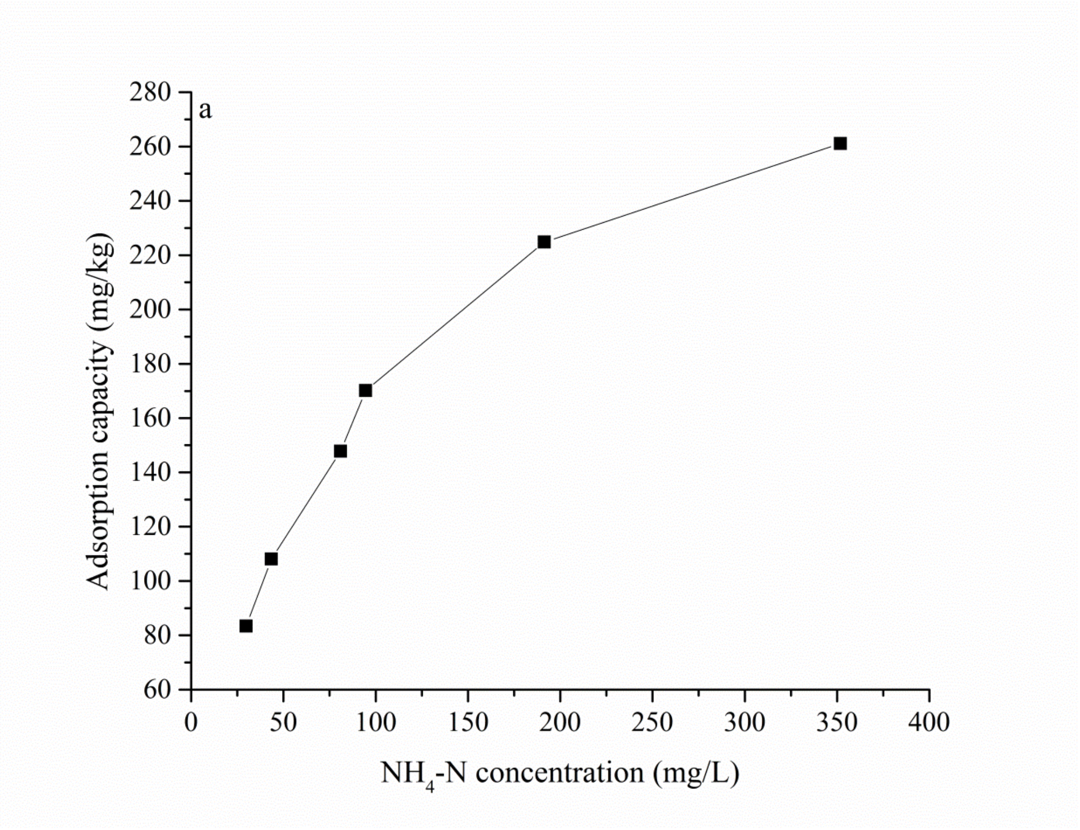

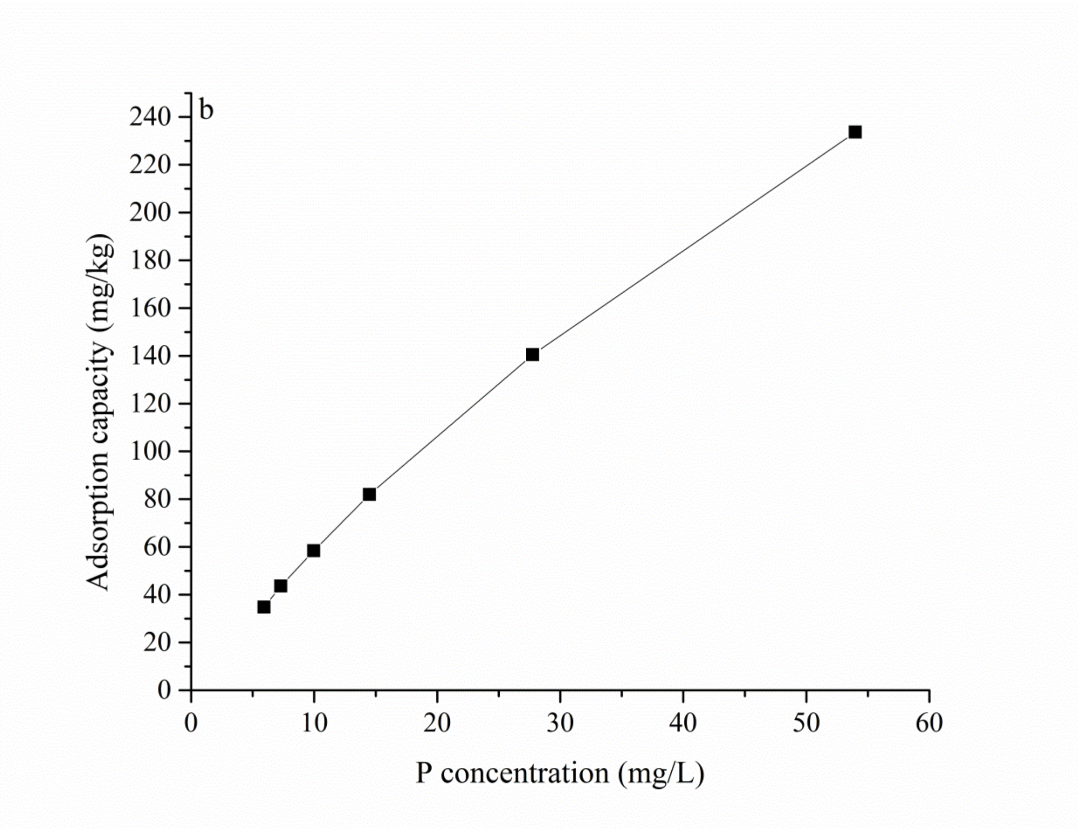

Figure S2 | Soil adsorption isotherms for (a) NH_4-N and (b) P with wastewater application.
